# Accompanimeter 1.0: creation and initial field testing of a tool to assess the extent to which the principles and building blocks of accompaniment are present in community health worker programs

**DOI:** 10.1080/16549716.2019.1699348

**Published:** 2019-12-12

**Authors:** Hector Carrasco, Harriet Napier, David Giber, Stephanie Kang, Mercedes Aguerreberre, Matthew Hing, Vinicius Siqueira Tavares Meira Silva, Mariana Montaño, Henry Perry, Daniel Palazuelos

**Affiliations:** a Doctor of Public Health Program, Harvard T.H. Chan School of Public Health, Boston, MA, USA; b Ciencias Básicas, Escuela de Medicina y Ciencias de la Salud del Tecnológico de Monterrey, México City, México; c Department of International Health, Johns Hopkins Bloomberg School of Public Health, Baltimore, MD, USA; d Community Health Systems, Partners In Health, Boston, MA, USA; e Department of Global Health and Social Medicine, Harvard Medical School, Boston, MA, USA; f Department of Maternal Health, Partners In Health (Compañeros En Salud), Ángel Albino Corzo, Chis, México; g David Geffen School of Medicine at UCLA, Los Angeles, CA, USA; h Family Medicine, Maria do Socorro Silva e Souza Family Clinic, Rio de Janeiro, Brazil

**Keywords:** Community health workers, quality improvement, the Accompanimeter 1.0, Partners In Health, primary health care

## Abstract

**Background**: The strategic incorporation of community health workers (CHWs) into health system strengthening efforts is recognized as a critical and high-value approach for meeting the Sustainable Development Goals established by the United Nations in 2015. How to best build CHW programs, however, is prone to a wide variety of opinions and philosophies, many of which are often externally imposed. Partners in Health (PIH) is a non-governmental organization that pioneered an approach to healthcare system strengthening, called accompaniment, in which CHWs play a key role. Learning from PIH is a critical first step in replicating the organization’s achievements beyond PIH. As such, PIH has developed a tool, referred to as the ‘Accompanimeter 1.0,’ that serves to evaluate existing CHW programs and guide adjustments in programming.

**Objective**: To provide a standardized approach for defining, assessing, and implementing accompaniment in CHW programs using a tool called the Accompanimeter 1.0.

**Methods**: Development of this tool included three stages: (1) desk review of literature relevant to the work of CHWs globally, (2) discussions among colleagues and initial field testing, (3) feedback from colleagues who are experts in community health and in the principles of accompaniment.

**Results**: Three core principles of accompaniment in a CHW program were identified: **professionalization, CHWs as bridges to institutional strength, and community proximity**. These core principles direct five thematic areas that are found in successful CHW programs: **Partnering** (co-creating engagement with a continuous and intersectoral dialogue to improve the program); **Choosing** (identifying the right people for the right job); **Educating** (building CHWs´ capacity); **Incentivizing** (enabling CHWs to perform their work without financial sacrifice); **Supervising** (mentoring CHWs for personal growth).

**Conclusions**: The Accompanimeter 1.0 can serve as a helpful tool for CHW program implementation and policy decisions that maximize system-side inputs, community engagement, and support for individuals with medical issues.

## Background

Community health workers (CHWs) are recognized by governmental and non-governmental stakeholders as critical contributors to strong and effective health systems [1]. Yet, how CHWs should be incorporated into healthcare-delivery structures has been subject to a wide variety of opinions and philosophies; for example, CHW programs targeting minor improvements in care-seeking behavior [2] or reductions in morbidity and mortality for a pre-specified sub-section of the population are often pitched as stand-alone, cost-effective models that health systems should replicate [3]. Furthermore, few frameworks provide operational or implementation guidance on how to make design choices; for example, the Alma Ata Declaration placed community health at the center of primary health care but did not offer advice on how a successful CHW program should be designed, implemented or evaluated. Intensified health inequity and imbalanced access to curative and preventive care in low-resource settings [4] demand alternative visions and methodologies. If ambitious health-related goals (such as UHC and the SDGs) are to be met, health systems must replace short-term and vertical donor-driven programs with truly functional CHW models that are strategically integrated into public primary health care systems.

Partners in Health (PIH) is a Boston-based non-governmental organization that has worked for over three decades across more than ten countries (such as México, Peru, Haiti, Russia, Rwanda, Malawi, Sierra Leone, Lesotho, Liberia and Navajo Nation) to produce some of the most notable health outcomes in global health [5]. The PIH approach to programmatic design and implementation is referred to as ‘accompaniment.’ Using this approach, PIH has demonstrated unprecedented cure rates of extensively drug-resistant TB (XDR-TB) and excellent clinical outcomes in Peru [6–8], high retention of treatment among persons with HIV in Rwanda [9,10], and some of the highest rates of clinical control documented in impoverished communities in Mexico for diabetes and hypertension [11], among others.

### Accompaniment in a community health worker program

Accompaniment in a CHW program is a vulnerability-oriented approach taken to ameliorate harmful social arrangements to improve health [5]. Harmful social arrangements include lack of access to effective and quality healthcare, food insecurity, multidimensional poverty, vulnerability, and stigma that limit one’s ability to live in a healthy environment, practice healthy behaviors, or reap the benefits of healthcare. CHW programs that promulgate the notion of accompaniment are uniquely capable of addressing these limitations by aiding in the transition of harmful economic, social, and political determinants of health into productive ones, and thus empowering the individual [5].

Accompaniment, however, is not a static or single intervention at the onset of a CHW program’s design – rather, it is a flexible, responsive and iterative process. As an institutional practice, accompaniment requires health systems to listen and empathize with the populations they aim to serve, identify key vulnerability gaps in care, and fill those gaps with appropriately funded and effective health actions. An *accompaneer* (or CHW using the accompaniment approach; in French *accompagnateur*; in Spanish *acompañante*) is willing to continuously listen, stand, and walk in solidarity with patients as they seek wellbeing, mediating interactions between patients, communities, and medical care providers, while simultaneously unveiling and addressing the social, economic, and political forces behind the illnesses of his or her patients.

In this paper, we describe the development of a tool called the Accompanimeter 1.0. This tool aims to guide the repositioning or re-engineering of CHW programs in low, middle or even high-income countries from an accompaniment standpoint through intentional budgetary, partnership, impact measurement and management process decision-making.

## Methods

To develop the Accompanimeter 1.0, we first agreed on a common definition of ‘accompaniment’: accompaniment is both a philosophical stance and a rubric for programmatic design and implementation. Grounded in notions of social justice and human rights, accompaniment is a unique methodology for approaching, defining, and delivering community-oriented, person-oriented health actions [12,13]. Moreover, accompaniment responds to both ‘biological’ and ‘non-biological’ factors that cause illness by ensuring equal weight is given to the biomedical and economic, social, and political determinants of health when working with communities to improve their wellbeing. In other words, to accompany vulnerable individuals and their communities on their journey to wellness requires continued dedication until those being accompanied declare that they no longer require such support [12,13].

For this analysis, a ‘community’ is defined as ‘a group of people with diverse characteristics who are connected by social ties, share common perspectives, and engage in joint action in geographical locations or settings’ [14]. When advocating to ‘engage with the community,’ we envision a variety of activities focused on engagement with committees and/or selected leaders that serve to represent the community, as recommended by the Jamkhed model [15]. Detailing the nuances of these assumptions is beyond the scope of this paper, but they should be considered, if not embraced, by those using this tool.

After agreeing on these definitions, we conducted a literature review to identify similar tools before workshopping the first iteration and received feedback from peers. Finally, we tested the tool using semi-structured interviews with experts on Accompaniment and CHWs.

The lead author (HC) conducted a literature search in Medline via PubMed (2006–2016) using combinations of the following MeSH search terms: ‘community health worker,’ ‘village health workers,’ ‘barefoot doctors,’ ‘health care delivery’ AND non-MeSH search terms: ‘framework,’ ‘community-based accompaniment,’ ‘companion’, ‘accompaniment,’ and ‘model.’ Searches were performed in December 2016.

Second, the subsequent iteration of the Accompanimeter was envisioned and created by HC through informal conversations and debriefs with peers knowledgeable of CHW programs. This process allowed for the introduction of new ideas and adjustments, many of which were not meant to be traced. On this step, we also performed an initial field test of the tool on two existing community health worker programs in Mexico and Brazil. These programs were chosen by 1) their contrasts – one is a very large, government-run, and well-established CHW program and the other is a small and newly founded program led by an NGO –, 2) our familiarity with these programs, and 3) their documented impact on improving people’s lives [11,16]. Then, by convenience and availability, we selected three CHW program managers and eight CHWs in each program to perform semi-structured interviews (managers) and focus groups (CHWs). Additionally, to rank each program, we also performed one focus group with nine community members in each program. Our team then compiled field notes and performed within-case analysis, which involved writing up and summarizing feedback from end-users related to the first iteration of the Accompanimeter. End-users reported to our team feedback on three main categories: usability/comprehensibility, completeness/accuracy, and appropriateness. From these field notes, a general consensus was reached by team members on the key themes and issues, which was then incorporated into the Accompanimeter second iteration. (See supplementary material for a description and ranking of the programs using the latest iteration of the Accompanimeter 1.0).

Next, we approached 17 identified CHW experts within and outside PIH based on 1) their experience with CHW programs, 2) their unique familiarity with the accompaniment approach, and 3) international work on community health, and requested their review of the tool. We aimed and achieved a balance in gender and 70 percent of the participants were U.S., based (and the rest in Latin America and Haiti). Of the 17 experts, ten agreed to participate in semi-structured interviews. The other seven submitted feedback in writing. In advance of the interviews, all expert advisors were provided with the second iteration of the Accompanimeter and a brief document explaining the goals of the tool. Between June 6^th^ and July 11^th^, 2017, semi-structured interviews were administered over the phone and transcribed to ensure the fidelity of the recommendations given. Interview questions were separated into five categories: 1) feedback about the tool, 2) best practices on accompaniment, 3) skills of an effective *accompaneer*, 4) personal experiences with the concept of accompaniment in programmatic design, and 5) suggestions for additional resources. Experts’ responses were then compiled into a summary document and assessed for key themes and final tool suggestions. If some comments or insights from the experts were found to be vague or unclear, the experts were contacted for a second interview, to reduce the potential of misunderstanding their feedback. Next, the research team reviewed and discussed the notes from all interviews, and synthesized suggestions for a semi-final iteration (not shown). This semi-final iteration was then re-sent to participants for verification and solicitation of any final revisions. The tool (as shown in Table 1 and Figures 2–5) was then updated to its final iteration (version 1.0).

**Table 1.** Accompanimeter 1.0 (final iteration 3 of 3).

Building Block	Stage	Definition	Example
**Top-Down (Maximizing System-Side Inputs)**			
**Supervising** and mentoring personal growth	Inadequate	Non-existent, infrequent, punitive, and/or data collection-oriented supervision.	The supervisor appears a couple of times a year to fill out a task checklist and chastise the CHW if the tasks are not achieved
	Growing	Frequent but perfunctory supervision. No recognition of career pathways, personal development, or amendments to improve care provision techniques.	The supervisor appears regularly to give support and feedback, and to encourage the CHW to do his/her best in the work.
	Aspiring	**Humanized supportive supervision** The job is doable and fair, structures exist to mentor and support CHWs so that they can perform as expected. Supervision is frequent, supportive, encouraging, and humanized. Ratios of CHWs per population covered, and Supervisors per CHWs are adequate to assure quality (i.e. both CHW and supervisor have the time to engage in quality interactions). There are opportunities for career advancement.	The supervisor meets on a regular basis with the CHW and is available in case of need. The supervisor not only gives feedback of activities and how to improve, but also guides CHWs toward meaningful professional and personal development, such as going to school, taking a certification course, and providing help in case the CHW is sick, etc. CHWs are supported before they are fired.
**Partnering** in co-creation	Inadequate	CHW tasks are purely technical (medical), designed as a means to ‘address human resources gaps’, and oriented towards ‘short-term cost savings’ for select conditions. There is no clear integration with other health system actors.	The CHWs only vaccinate children or address one disease vertically. If they find a person with another condition, they do not perform any action to help that person.
	Growing	CHWs tasks are integrated in clear ways with the duties of other actors and sectors within the health system. Tasks can be clearly mapped out as a disease-oriented care-delivery value chain. Tasks are largely technical (medical), and the scope of work, workflow and ratios (e.g. CHWs per population covered) are designed to ensure completion of such technical (medical) tasks	The CHW only sees NCDs patients and has neither time nor resources to support a patient with an acute illness; the CHW does not go out and find new cases of a disease that recently arrived to the community.
	Aspiring	**Community work, not just health work** The health system is built to progressively realize universal health coverage. CHWs perform tasks that adequately cure/control disease (sometimes in conjunction with tasks performed by other team members). CHWs are bridges between the community and health system, and not islands of limited interventions. CHW tasks are thoughtfully integrated with the work of nurses and doctors, as well as with other sectors such as education, finance and social development. Their scope of work, workflow, and ratios (e.g. CHWs per population covered) permit the performance of not only technical (medical) tasks but also empathetic (non-medical) tasks (i.e. social support, advocacy, community work). Structures exist for CHWs to have a voice in ongoing program reform.	The program intentionally invests in mechanisms that empowers the CHW to amplify community voice, advocate for individual patients, and perform proactive case finding. CHWs are not simply given a medicine box and asked to do ‘the best they can’ with a limited toolkit.
**Incentivizing** by enabling	Inadequate	Neither financial nor non-financial benefits are provided to the CHWs. The ‘Spirit of Volunteerism’ is considered the primary motivation.	The CHWs perform their duties if, and when, they have the will and the resources to do so but receive no financial support to facilitate or appreciate this work.
	Growing	Some financial or non-financial benefits are provided as a source of motivation, but not to enable or empower CHWs.	The CHWs receive a food package to acknowledge their efforts in performing their duties.
	Aspiring	**Professionalization for long-term retention** The CHW’s salary allows them to dedicate their full time and attention on community health work because it provides for all their material needs. Financial and non-financial benefits even facilitate a pathway out of poverty over the long-term.	The salary is at least minimum wage for a full time job. The local poverty line is defined and a life span of work will move a CHW above the poverty line. CHWs also receive benefits such as health care, vacation time, retirement support, etc.
**Choosing** the right people for the right job	Inadequate	CHWs are selected in a non-transparent way. There is significant risk of nepotism, and CHWs do not necessarily represent the people they serve.	A community leader decides who becomes the CHW without community input, or the community decides based on the only available people who want to become CHWs, even if the candidates do not fulfill health system and community criteria.
	Growing	CHWs represent the community and health system simultaneously. The selection process is transparent, with clear criteria oriented towards benefitting both the community and the health system.	The CHWs are perceived as the most skilled persons in the community. Minorities may not be represented in the program. The program is likely to select already empowered people.
	Aspiring	**CHWs strongly represent the health system, and all the communities** The selection process is transparent, and criteria for selection are clear. There are mechanisms for recruitmenting and supporting people from vulnerable populations (i.e. women, ethnic minorities, etc.).	Communities nominate candidates from a large and diverse pool during a community meeting. Program leaders then list the best candidates based on clear criteria (i.e. ability to walk to house visits, basic literacy, etc.), but also provide special supports for recruiting and preparing CHWs from vulnerable minority groups (such as literacy courses, job training, and skills training, etc).
**Education** for capacity building	Inadequate	The program provides training once or a few times, and the training is predominantly technical.	One baseline training is administered as a series of PowerPoint slides and/or a training session in which only the facilitator speaks. No continuing education is provided.
	Growing	Training is well-structured, responsive to needs, ongoing, and competency based. Training balances technical and empathic skills.	The CHWs receive ongoing training to perform their duties, but without a contextual and sociological frame to effect community change.
	Aspiring	**Training for transformation** The education offered builds over time and is clearly linked to a professional development pathway. Training integrates technical, empathic, and social justice skills, resulting in a CHW who is a “liberator,” not a “lackey.” [27]	The training helps CHWs grow not only in their role as workers performing the job assigned to them by the health system, but as partners and advocates to achieve positive social change in the community.
**Bottom-Up (Maximizing Patient-Community Agency)**			
**Supervising** and mentoring personal growth	Inadequate	In general, the community has little to no engagement in determining supervision structure and measures.	The community is aware of the presence of CHWs and receives the CHWs’ services, but is not involved In monitoring or feedback.
	Growing	The community has some input in the program, which might include the election of community-based supervisors, community councils, performing patient satisfaction surveys or community meetings to discuss or approve CHWs activities. However, community-based input rarely stimulates program alterations or improvements.	A select member of the community is identified as a field-based supervisor and is tasked with predetermined supervisory duties without much agency to work with community input to improve the program.
	Aspiring	**Local truth: Favoring EQ (emotional quotient) as much as IQ (intelligence quotient)** Structures exist to assure that community members’ opinions are captured, heard and acted upon so that CHWs behavior is truly patient-centered and empathic. Major violations of patient trust are grounds for termination (i.e. violence, coercion, abuse, breaking confidentiality)	Patients surveys are collected by CHW supervisors on regular intervals and incorporated in regular feedback sessions. Community health committees are an integral part of decision making. There is a simple way for patients to report violations (and have their identity protected).
**Partnering** in co-creation	Inadequate	The program does not actively address the social environment in which CHWs operate. It neither attends to social determinants of illness nor meets ‘demand side’ barriers. The program exhibits no attempts to collaborate with other sectors.	The program performs and delivers on technical tasks but does not attend to other relevant factors that influence health.
	Growing	The program recognizes the need to address the social environment in which CHWs operate. The program attends to social determinants of illness and meets demand-side barriers, but the budget for the program and the CHWs scope of work does not equally reflect this recognition. The program addresses a few social determinants of health, but this is not considered in the budget.	A CHW might refer a patient to a social program, but this is not a crucial part of his/her duties. A policy exists that sick patients should get nutrition support (such as a TB, AIDS or cancer patient), but in reality, there is no budget allocated to food procurement.
	Aspiring	**Going beyond the clinic, and the clinical** The program actively addresses social determinants of illness and posits demand-side barriers to accessing healthcare, or achieving good health outcomes, as major priorities. A significant portion of the budget is dedicated to such efforts. Collaboration with other sectors is prioritized as a mechanism to achieve this goal.	The program includes transport vouchers, food programs, housing programs, water programs, jobs/skills programs, income support, or educational programs in their plans. This is often done by collaborating with other government agencies, other NGOs, cooperatives, etc.
**Incentivizing** by enabling	Inadequate	The community does not recognize the value of the CHW program to improve or maintain health. The community does not invest in generating social capital (enabled people to take care of others) nor support the CHWs’ work.	The community knows about the program, but is not invested in its success. If the program were to disappear, it would not cause the community much or any distress.
	Growing	The community recognizes the added value of the CHWs program to improve or maintain health, but it is not involved in a structured process to support the CHWs and to generate capital that will contribute to their motivation.	The community incentivizes the CHWs with gifts but is not part of the design and implementation of the program. For example, there are no community meetings to recognize the efforts of CHWs.
	Aspirational	**This is how we value you** Structures exist that help community members show appreciation to CHWs in the manner of their choosing (verbal, material, financial). This is not an obligation for community members, but rather reflects the trust and value being generated by the community health system.	The community sets salary scales, augments salaries with money or gifts, enables supports (i.e. meals, housing or water) when CHWs are in the field, publicly recognizes CHWs with award ceremonies, and/or honors CHWs during other key community events,
**Choosing** the right people for the right job	Inadequate	The community does not participate in developing the job description, in recruiting, or in selecting the CHWs. The entire process is managed and executed by the health system or by an institution external to the community.	Implementers external to the community carry out the entire process of recruiting CHWs without any community input.
	Growing	The community participates in the recruitment and selection processes of the CHWs; however, their suggestions are ultimately less decisive than those of program implementers, managers, funders, or policy makers.	Community members can nominate CHWs but the final decision on hiring, and the criteria used for hiring, is executed by the program implementers without additional community input.
	Aspiring	**The community’s voice and vote** There is a dedicated and funded mechanism that engages community members in contributing to job description development, nomination, and selection of CHWs.	Community members are in charge of nominating CHWs but are also tasked with working closely with program implementers to decide the characteristics of the ideal CHWs.
**Education** for capacity building	Inadequate	Community members do not participate in developing the curricula for training CHWs. Community knowledge (local wisdom) and local culture are dismissed.	A local term for a symptom is categorized as not scientific, wrong or dangerous by the CHWs’ training curricula.
	Growing	Community members are informed of the curricula and training of CHWs, but their input is not requested. Community knowledge and local culture are acknowledged, discussed, and respected by the CHWs’ training curricula but not incorporated into the program.	The program is not against local wisdom or building on the knowledge that the CHWs bring to the training seasons, but this is not actively encouraged and introduced in the curricula.
	Aspiring	**Local wisdom** Processes exist to actively engage with, discuss, AND incorporate local knowledge into the CHW curricula and training.	Community members can participate in building the training curricula for the CHWs, and they are enabled to do so (e.g. with a stipend). Likewise, new information is built on top of the existing knowledge that the CHWs bring to the sessions, capturing the richness of the lived experience in their unique context.

In short, the literature review supplied best practices in CHW programs, the informal conversations and initial field testing refined the tool further and semi-structured interviews with experienced individuals in Accompaniment enhanced and polished the tool to its third and final iteration.

## Results

### Results of the literature review

**Figure 1.**
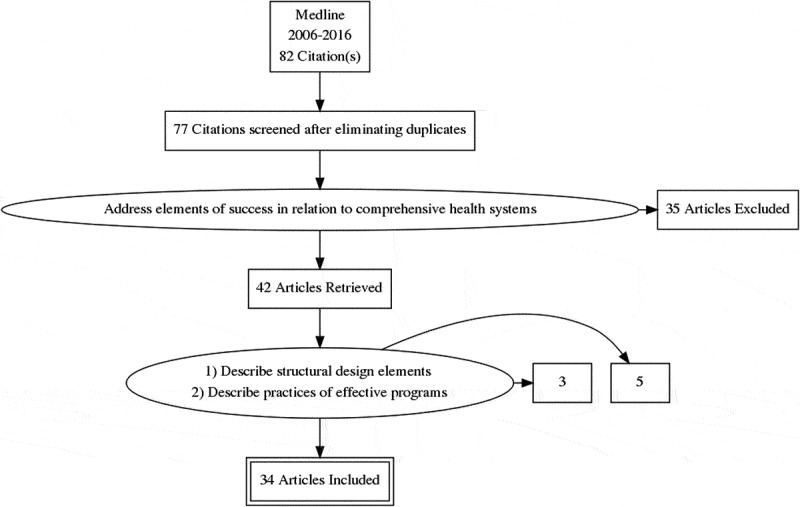
Review on frameworks describing elements of success in CHW programs.

**Figure 2.**
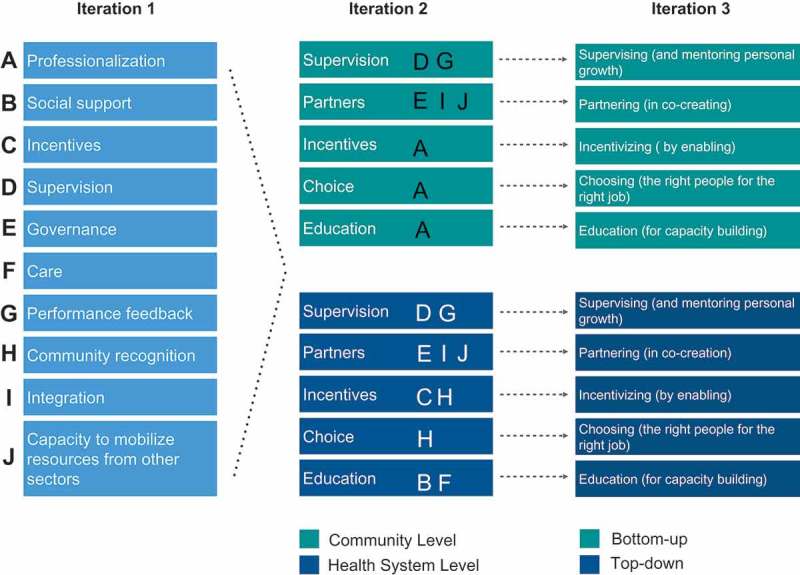
Accompanimeter evolution.

We identified 82 abstracts and reports that mentioned the elements of a successful CHW program while offering a theoretical framework. From these, five abstracts were excluded because they were duplicative. From the 77 documents remaining, 35 more were excluded because they referred to isolated programs not integrated into comprehensive health systems. The 42 papers and reports remaining were organized into two categories: (1) those that described the structural design elements of effective CHW programs and (2) those that described the practices of effective CHW programs. From these two categories and based on improvement of clinical outcomes and CHW satisfaction, we selected 34 papers and reports to inform the first draft of the Accompanimeter (see Figure 1 and supplementary files for a complete list of the papers selected). This first iteration compiled the ten attributes of an effective CHW program most mentioned in the literature (see Figure 2, iteration 1 of 3). During this process, the research team encountered several established frameworks, including: The Community Health Worker Assessment and Improvement Matrix (CHW AIM) [17], 5-SPICE [18], The Program Functionality Assessment Toolkit [19], and The CHW Principles of Practice [20].

### Results of informal conversations and during the initial field test

The 5-SPICE framework emerged as the most parsimonious yet still comprehensive tool for program design. This tool outlines the crucial elements – along with their positive and negative interactions – of successful CHW programs. The elements of the 5-SPICE framework include Supervision, Partners, Incentives, Choice, and Education [18]. CHW program managers praised how the 5-SPICE key elements, ease of use and guidance on how to strategically address challenges and visualize different components of the program. Other frameworks contained a higher number of elements which may lead to them being less memorable and more difficult to comprehend the scope of some elements and interactions with others. The 5-SPICE framework was expanded upon to develop the accompaniment tool and this was called iteration 2 of 3 of the Accompanimeter (see Figure 2).

During the semi-structured interviews, three common topics emerged consistently. 1) As opposed to 5-SPICE, the Accompanimeter 1.0 should have more comprehensive domains instead of focusing on narrow human resources’ terminology, 2) the new tool should include levels of performance with concrete examples for each domain, and 3) the Accompanimeter 1.0 should include an implementation guide so that it can become a pedagogical tool and also invite an enlightened dialogue among all the different stakeholders of the CHW program. Iteration 3 of 3 of the tool considered these recommendations along with other minor suggestions such as more positive terminology and changing the accompaniment’s stages from ‘none, moderate, and excellent’ to ‘inadequate, growing and aspirational’. The conceptual idea of the Spidergrams was based on the work of Baatiema and colleagues [21]. We decided to use two approaches when evaluating a CHW program: the bottom-up and the top-down. The former shows how to maximize health system inputs and support better a CHW program and the latter displays benchmarks in different building blocks to maximize patient-community agency. We believe the two approaches provide complementary perspectives for designing an effective CHW program.

Through the literature search, informal discussions, initial field testing, and semi-structured interviews, three principles emerged as critical to making accompaniment actionable in a successful CHW program.

1. ***Professionalization***: Programs that aim for professionalized CHWs commit to long-term investments in their CHWs to ensure their success and retention as core members of effective care delivery teams. This commitment begins with:
  - Adequate recruitment and hiring based on candidate interest in the CHW responsibilities and candidate potential to excel in the role.
  - High-quality, ongoing training to provide CHWs with the knowledge and skills necessary to perform their roles with confidence.
  - A fair package of incentives commensurate with the scope of work to ensure that CHWs can commit the time needed for the job and achieve the economic freedom required to focus on their duties.
  - Supportive supervision with ongoing mentorship that facilitates personal and professional growth, and fosters active problem solving and sustained performance with excellence and fidelity.
  - Clear career-development pathways that offer opportunities for high-performers to grow professionally.
2. ***CHWs as Bridges to Institutional Strength***: Programs that offer ‘institutional strength’ to community health programs position CHWs not as islands of care but rather as bridges to a functioning health system. In that way, the tasks that CHWs perform contribute to the value chain of care delivery and complement the work of nurses, doctors, pharmacists and other healthcare providers, which is expected to produce better outcomes. While CHWs strengthen health systems, they can only be as strong as the systems that support them. This results in a feedback loop. Practically, this means that the CHW programs cannot be strong if funded alone while the larger health system is comparatively underfunded.
3. ***Community proximity***: Enthusiasm for data demonstrating the CHWs’ capacity to achieve reductions in mortality at a fraction of the cost of other health providers can encourage health systems to convert the CHW into an overly simplified, highly-medicalized mortality-reduction agent. Our alternate and improved model positions the CHW as a colleague who can represent the health system in the community and the community in the health system. There is wisdom in the collective experiences of impoverished communities. CHWs can be the ‘Rosetta stone’ for translating that knowledge into action when designing, adapting and improving health and social services. A health system that values proximity with the community does not assign a CHW to work only inside a health center, but rather empowers a CHW to be out in the community, speaking to people openly (always honoring confidentiality and privacy agreements) and experiencing what they are truly living. Such a health system also fosters concrete and functional mechanisms for channeling and accounting for community experience (i.e. a community health committee and/or close partnership with community leaders).

### The analytical framework

**Figure 3.**
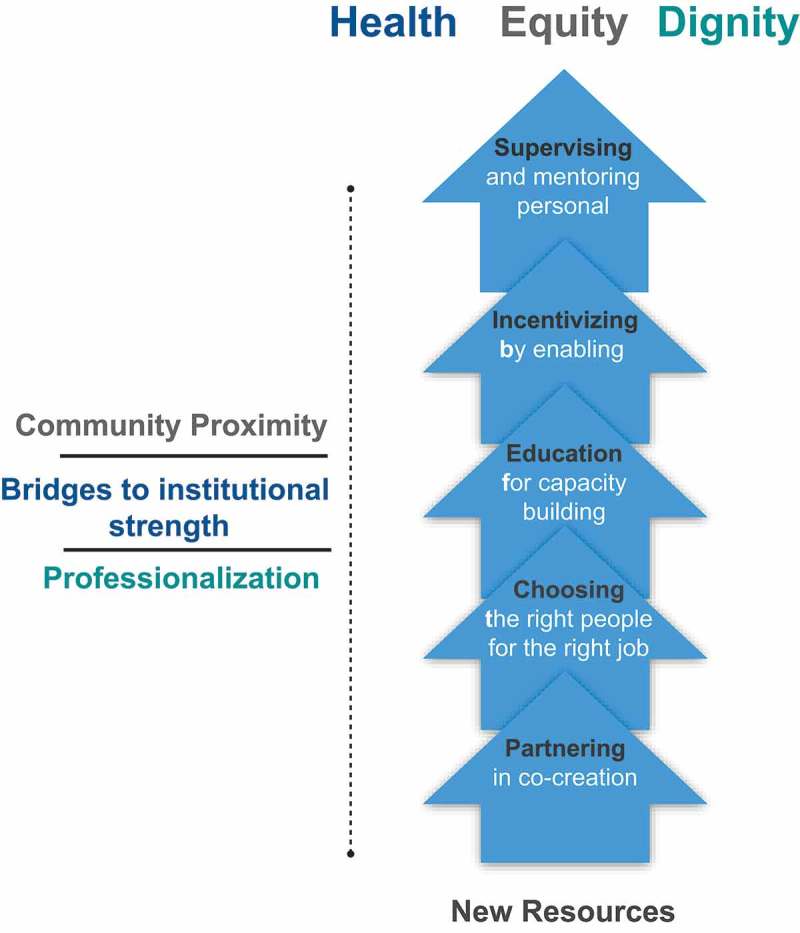
Three principles and five building blocks of accompaniment in CHW program.

The analytical framework is based on the notion that five thematic areas (Supervising, Partnering, Incentivizing, Choosing and Education) serve as the building blocks or platform on which the principles of an accompaniment-based CHW program can mature. Professionalization, CHWs as bridges to institutional strength, and community proximity are the three core principles of the PIH CHW model [5]. When these principles are present, and the building blocks tuned at the aspiring level, the effects can be synergistic and transformative. The ultimate goal of this framework is not only to reduce morbidity or mortality, or create job opportunities, but to achieve the greatest possible level of health, dignity, and equity for the populations served, as well as to generate trust and confidence in the health system (see Figure 3). As defined by the WHO in 1948, health is ‘a state of complete physical, mental, and social well-being and not merely the absence of disease’ [22]. True dignity in health requires tangible examples that demonstrate esteem for all people and are driven by a recognition of a common humanity [23]. Finally, equity is intentionally more biased than fairness as it strives actively to reconcile the structural factors that prevent some individuals or groups from achieving their greatest potential [24,25]. Addressing health with an explicit dignity and equity agenda is driven by a recognition that minor reductions in mortality are insufficient to truly break the link between poverty and disease.

We believe that the three principles and five building blocks enable programs to help health systems best reach the three dimensions of Universal Health Coverage: (1) coverage, (2) scope of services, and (3) financial protection. In addition, they add a very important focus on equity, highlighting the imperative for well-designed CHW programs to reach the most vulnerable [26].

## Discussion

### The Accompanimeter 1.0 versus other tools

Many frameworks offer useful insights on how to design and improve a CHW program. The Community Health Worker Assessment and Improvement Matrix (CHW AIM) (USAID) is comprehensive and well-supported from an academic standpoint, and it offers a detailed pathway to assess a CHW program; however, it does not explain how the different elements interact with each other and lacks examples on how these elements are implemented in real life [17]. The Accompanimeter 1.0, in turn, offers examples based on PIH’s field experience.

The Program Functionality Assessment Toolkit (Core + World vision) contains a detailed explanation of each programmatic component and is easy to understand, but it lacks evidence on how the components were chosen nor does it offer any practical recommendations to implement it via real world examples [19].

The CHW Principles of Practice is short, concise, visual, and offers a few practical recommendations, but lacks examples of how the different principles look in the field, nor does it highlight any potential interactions among principles [20].

And finally, the 5-SPICE framework (PIH) is comprehensive, concise and shows examples of interactions among elements, but it does not mention how to implement it, nor does it include different benchmarking levels for each programmatic element. Also, it fails to convey system-side and community-side perspectives on each component with practical examples [18].

Nevertheless, the Accompanimeter 1.0 can be considered an extension of 5-SPICE since it uses the 5-SPICE structure to map out what a CHW program built around the values of accompaniment could look like. The practical applications of each tool, however, are quite different: 5-SPICE is most useful for initiating an exploratory conversation about how a program is structured; the 5 × 5 tables described in that paper offer an open-ended methodology to explore what does and does not work in a specific program. The Accompanimeter 1.0, on the other hand, is far more normative about what should be aspired for in a community health program that is guided by the accompaniment approach.

### Using the tool

The Accompanimeter 1.0 should be used in a collaborative fashion with different stakeholders who are invested in the CHW program at any point in its development or maturation. The process of implementing the tool is iterative; at various points in the program implementation of the CHW program, managers, health personnel, program supporters, CHWs, and community members should carry out an assessment, and appropriate action can be taken.

**Figure 4.**
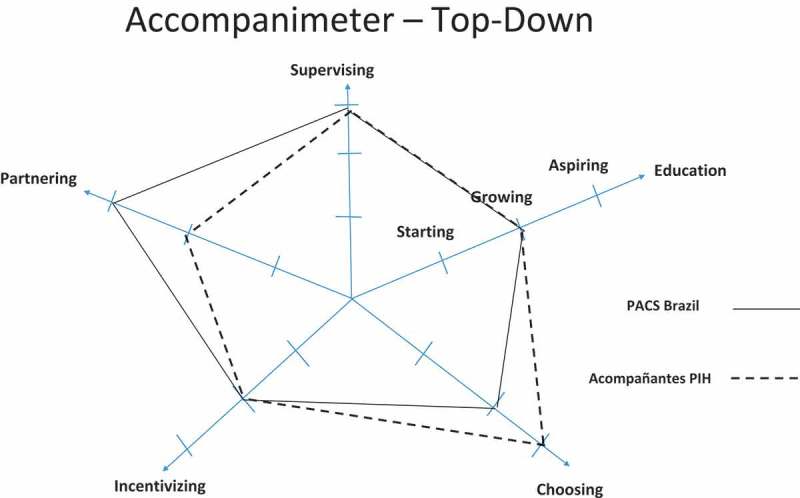
Accompanimeter 1.0.

**Figure 5.**
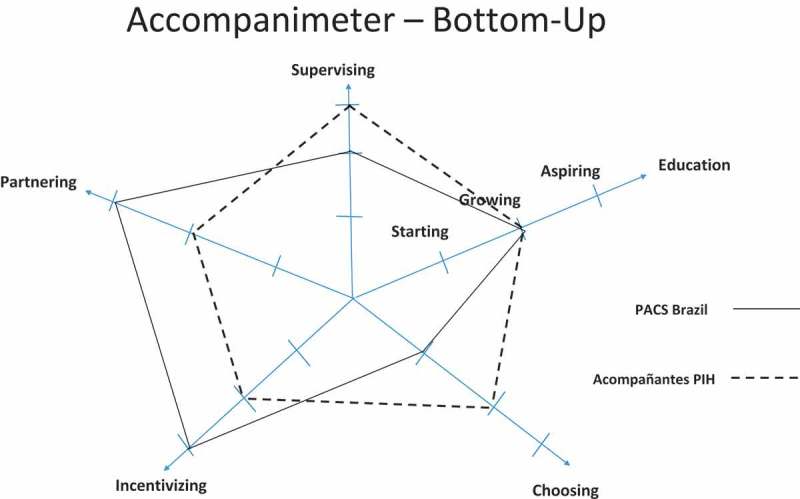
Accompanimeter 1.0.

For existing programs, we recommend the following steps for utilization of the tool:

1. **Assess**: Each program should use Table 1 to conduct an honest assessment of the current program. To choose the stage on each building block, each participant should read the definition and the examples offered in Table 1. This analysis will lead participants to reflect on the program as it currently exists, and they should be encouraged to use these thoughts to briefly formulate a statement on their view regarding the current program’s priorities.
2. **Review ratings**: During a CHW program stakeholders meeting, a facilitator should assemble, average, and display the ratings (using the Spidergrams in Figures 4 and 5), present the program priorities statements offered by all the participants, then discuss the differences noted. After a facilitated debate and discussion, the team should garner consensus on areas for improvement as identified by the tool.
3. **Brainstorm**: A facilitator should then lead the team through a brainstorming session to identify possible approaches for filling identified gaps in an encouraging and respectful manner. This exercise should result in two lists:
  - Possible solutions that are feasible to implement.
  - More intensive issues to address (i.e. issues that mght require additional resources to be resolved).
4. **Plan**: The team should order the solutions based on feasibility and then develop action plans for addressing them. This may include re-writing agreements with existing partners, looking for new resources, hiring new managers, allocating resources to new areas, re-defining indicators, and so forth. It is crucial that the stakeholders assigned to lead the implementation of a particular solution are carefully selected; for example, community members might be more effective at reengaging community-level committees whereas the program manager may be better positioned to engage new potential donors.
5. **Implement**: Once action plans are determined, the teams can take the action plan to the field in a collaborative manner by engaging multiple stakeholders in the implementation process. As many potential changes are expected to emerge, it is important to list them and strategically address them over time, instead of simply stopping once a pressing challenge is solved. In addition, the team should determine a mechanism of follow-up and establish systems for accountability in the Accompanimeter 1.0 implementation process.

In Figures 4 and 5 we show an assessment of the PACS program in Maria do Socorro Silva e Souza Family Clinic (Brazil) and of the Acompañantes program in Laguna del Cofre, PIH México. We also provide the participants’ insights and quotes that allowed us to rank the program in each building block (see supplementary material).

### Applications of the tool

The Accompanimeter 1.0 can serve policy makers and program implementers in low, middle and high income countries leading small, medium or large CHW programs to:

- Identify design or implementation gaps that limit a CHW program’s potential to achieve ambitious clinical and/or non-clinical health outcomes.
- Illuminate the pathway a CHW program can follow to move from a short-term healthcare delivery intervention to a long-term integrated equity-focused solution.
- Initiate a dialogue on how to build or rebuild community-level health care systems that are well-balanced and responsive to the needs and goals of both the healthcare system and the community.

The Accompanimeter 1.0 tool has several distinctive features:

1. It is *reflective* of the accompaniment values across PIH;
2. It is *simple* and allows CHW program stakeholders to analyze their programs with a single and cohesive model;
3. It is *visual*, which assists greatly in comparing different programs, assessing improvements over time in a single program, and communicating the results of the analysis to lower-literacy stakeholders such as CHWs and community members in a multi-dimensional fashion;
4. It is *comprehensive* and combines evidence-informed criteria with a wealth of experts’ experience;
5. It *demonstrates* how an aspiring accompaniment-based program can be modeled; and
6. It is uniquely focused on *equity* in clinical outcomes, which is defined as concrete and measurable benefits experienced by the poorest or most vulnerable.

### Limitations

No single tool can be everything for every environment, organization, and program. The Accompanimeter 1.0 was created primarily to promote the values of accompaniment as central to an impactful CHW program. Though based on decades of experience, many measures of the tool are still subjective. As most key informants were affiliates of the PIH network, non-PIH programs may encounter challenges in using the tool that were not uncovered during the discussions. In addition, most of the experts are based in the U.S. which might have added some biases to the tool. Still, the specific advice in this tool may still be adapted as further research or experience suggest better practice. As such, this tool should not be construed as block advice to be implemented without further analysis, but rather as a collection of normative statements that users can interpret for implementation in their unique contexts.

## Conclusion

The Accompanimeter 1.0 is a unique tool that enables community health policy makers and program implementers to put into practice the three core principles of accompaniment (professionalization, CHWs as bridges to institutional strength, and community proximity). Furthermore, this tool also offers CHW program stakeholders a participatory process to assess, discuss, and modify, as needed, the core building blocks of their CHW program to effectively reorient towards a clear path to health, dignity, and equity advancements.

## Paper context

CHWs programs have received greater attention with the advent of the SDGs. Our paper provides a guide for designing CHW programs around the values of an approach called ‘accompaniment,’ and supplies critical feedback around the essential elements that drive impact at community and health system levels. The Accompanimeter 1.0 tool can help programs achieve better health outcomes with a focus on equity, enhance stakeholder participation in the program, and more effectively pursue and use resources.
