# Supplementary material for: Accompanimeter 1.0: creation and initial field testing of a tool to assess the extent to which the principles and building blocks of accompaniment are present in community health worker programs
Source: Glob Health Action. 2019 Dec 12;12(1):1699348. doi: 10.1080/16549716.2019.1699348 (PMC6913655; doi:10.1080/16549716.2019.1699348)
Supplement: Supplemental Material [file ZGHA_A_1699348_SM2122.docx]

**Supplementary material**

*Note:* This material can either be made available as supplementary material online only, or it can included as part of the published paper, depending on the editors’ preferences.

**Final list of the papers selected from the literature review [^1–33^ ^34^]**

1. Crigler, L., Hill, K., Furth, R. & Bjerregaard, D. A Toolkit for Improving CHW Programs and Services. 146 (2011).

2. Palazuelos, D. *et al.* 5-SPICE: the application of an original framework for community health worker program design, quality improvement and research agenda setting. *Glob. Health Action* **6**, 19658 (2013).

3. Stone, J. R. & Parham, G. P. An ethical framework for community health workers and related institutions. *Fam. Community Health* **30**, 351–363 (2007).

4. Mutale, W. *et al.* Application of systems thinking: 12-month postintervention evaluation of a complex health system intervention in Zambia: the case of the BHOMA. *J. Eval. Clin. Pract.* **23**, 439–452 (2017).

5. Baatiema, L., Skovdal, M., Rifkin, S. & Campbell, C. Assessing participation in a community-based health planning and services programme in Ghana. *BMC Health Serv. Res.* **13**, 233 (2013).

6. Glenton, C. *et al.* Barriers and facilitators to the implementation of lay health worker programmes to improve access to maternal and child health: qualitative evidence synthesis. *Cochrane Database Syst. Rev.* CD010414 (2013) doi:10.1002/14651858.CD010414.pub2.

7. Gaudrault, M., LeBan, K., Crigler, L. & Freeman, P. Community Health Management Committee Assessment and Improvement Matrix (CHMC AIM) September 2015. 19.

8. Kowitt, S. D., Emmerling, D., Fisher, E. B. & Tanasugarn, C. Community Health Workers as Agents of Health Promotion: Analyzing Thailand’s Village Health Volunteer Program. *J. Community Health* **40**, 780–788 (2015).

9. Pinto, R. M., da Silva, S. B. & Soriano, R. Community health workers in Brazil’s Unified Health System: a framework of their praxis and contributions to patient health behaviors. *Soc. Sci. Med. 1982* **74**, 940–947 (2012).

10. Saprii, L., Richards, E., Kokho, P. & Theobald, S. Community health workers in rural India: analysing the opportunities and challenges Accredited Social Health Activists (ASHAs) face in realising their multiple roles. *Hum. Resour. Health* **13**, 95 (2015).

11. Rachlis, B. *et al.* Community Perceptions of Community Health Workers (CHWs) and Their Roles in Management for HIV, Tuberculosis and Hypertension in Western Kenya. *PloS One* **11**, e0149412 (2016).

12. Rosenthal, E. L. *et al.* Critical reflections on the role of CBPR within an RCT community health worker prevention intervention. *J. Ambulatory Care Manage.* **37**, 241–249 (2014).

13. Perry, H., Crigler, L. & Hodgins, S. *Developing and Strengthening Community Health Worker Programs at Scale: A Reference Guide and Case Studies for Program Managers and Policymakers*. https://www.chwcentral.org/developing-and-strengthening-community-health-worker-programs-scale-reference-guide-and-case-studies (2014).

14. Nzioki, J. M., Onyango, R. O. & Ombaka, J. H. Efficiency and factors influencing efficiency of Community Health Strategy in providing Maternal and Child Health services in Mwingi District, Kenya: an expert opinion perspective. *Pan Afr. Med. J.* **20**, 88 (2015).

15. Young, C. *et al.* Health professional and community perspectives on reducing barriers to accessing specialist health care in metropolitan Aboriginal communities: A semi-structured interview study. *J. Paediatr. Child Health* **53**, 277–282 (2017).

16. McCollum, R., Gomez, W., Theobald, S. & Taegtmeyer, M. How equitable are community health worker programmes and which programme features influence equity of community health worker services? A systematic review. *BMC Public Health* **16**, 419 (2016).

17. Goodman, R. M. *et al.* Identifying and defining the dimensions of community capacity to provide a basis for measurement. *Health Educ. Behav. Off. Publ. Soc. Public Health Educ.* **25**, 258–278 (1998).

18. Rabbani, F. *et al.* Inspiring health worker motivation with supportive supervision: a survey of lady health supervisor motivating factors in rural Pakistan. *BMC Health Serv. Res.* **16**, 397 (2016).

19. Zulu, J. M., Kinsman, J., Michelo, C. & Hurtig, A.-K. Integrating national community-based health worker programmes into health systems: a systematic review identifying lessons learned from low-and middle-income countries. *BMC Public Health* **14**, 987 (2014).

20. Kane, S. *et al.* Limits and opportunities to community health worker empowerment: A multi-country comparative study. *Soc. Sci. Med. 1982* **164**, 27–34 (2016).

21. Pessoa, V. M., Rigotto, R. M., Carneiro, F. F. & Teixeira, A. C. de A. [Meanings and methods of territorialization in primary health care]. *Cienc. Saude Coletiva* **18**, 2253–2262 (2013).

22. Kok, M. C. & Muula, A. S. Motivation and job satisfaction of health surveillance assistants in Mwanza, Malawi: an explorative study. *Malawi Med. J. J. Med. Assoc. Malawi* **25**, 5–11 (2013).

23. Katigbak, C., Van Devanter, N., Islam, N. & Trinh-Shevrin, C. Partners in health: a conceptual framework for the role of community health workers in facilitating patients’ adoption of healthy behaviors. *Am. J. Public Health* **105**, 872–880 (2015).

24. Morgan, A. U., Grande, D. T., Carter, T., Long, J. A. & Kangovi, S. Penn Center for Community Health Workers: Step-by-Step Approach to Sustain an Evidence-Based Community Health Worker Intervention at an Academic Medical Center. *Am. J. Public Health* **106**, 1958–1960 (2016).

25. Sabo, S. *et al.* Predictors and a framework for fostering community advocacy as a community health worker core function to eliminate health disparities. *Am. J. Public Health* **103**, e67-73 (2013).

26. Fitzgerald, T. M. *et al.* Program Implementation Approaches to Build and Sustain Health Care Coordination for Type 2 Diabetes. *Health Promot. Pract.* **18**, 306–313 (2017).

27. *Strengthening the performance of community health workers in primary health care: report of a WHO study group*. (World Health Organization ; WHO Publications Center USA [distributor], 1989).

28. Lunsford, S. S., Fatta, K., Stover, K. E. & Shrestha, R. Supporting close-to-community providers through a community health system approach: case examples from Ethiopia and Tanzania. *Hum. Resour. Health* **13**, 12 (2015).

29. Maulik, P. K., Tewari, A., Devarapalli, S., Kallakuri, S. & Patel, A. The Systematic Medical Appraisal, Referral and Treatment (SMART) Mental Health Project: Development and Testing of Electronic Decision Support System and Formative Research to Understand Perceptions about Mental Health in Rural India. *PloS One* **11**, e0164404 (2016).

30. WHO. *The World Health Report 2008 - primary Health Care (Now More Than Ever)*. https://www.who.int/whr/2008/en/ (2008).

31. Iwelunmor, J. *et al.* Toward the sustainability of health interventions implemented in sub-Saharan Africa: a systematic review and conceptual framework. *Implement. Sci. IS* **11**, 43 (2016).

32. Werner D. The village health worker--lackey or liberator? [proceedings]. World Hosp. 1978;14:32.

33. Kok, M. C. *et al.* Which intervention design factors influence performance of community health workers in low- and middle-income countries? A systematic review. *Health Policy Plan.* **30**, 1207–1227 (2015).

34. Covert, H., Sherman, M., Miner, K. & Lichtveld, M. Core Competencies and a Workforce Framework for Community Health Workers: A Model for Advancing the Profession. *Am. J. Public Health* **109**, 320–327 (2018).

**Initial Field Testing Sites: a brief contextual description**

Agentes Comunitários de Saúde in Brazil

The unique Brazilian public primary care model, known today as the Family Health Strategy (*Estratégia de saúde da família*), began as a program focused mainly on CHWs, and later evolved to include other healthcare personnel [1]. The CHWs program PACS (*programa de agentes comunitários de saúde*) began in the early 1990s, shortly after the promulgation of the current federal Constitution of Brazil, as one of the initial steps to rapidly implement a universal healthcare system, a fundamental part of the country’s welfare state [1].

Participatory health councils, a critical component of the Brazilian Health System, allow citizens to oversee the country's public health system (the Sistema Único de Saúde - Unified Health System or SUS). These councils exist at the municipal, state, and national levels, and are supplemented by a national conference on the Brazilian health system held every four years. To date, there are 5,562 municipal councils around Brazil [2].

According to current legislation, Brazilian CHWs are health system workers integrated into a multidisciplinary Family Health Team (FHT). Each team is composed of up to twelve CHWs along with at least one doctor, one nurse, and one nurse assistant. Each FHT is responsible for approximately 1,000-2,000 families, having more or less 3,500-4,500 people and each CHW is responsible for delivering specific health services (see table 5) to around 110 families [2].

PACS has achieved success in several arenas, with strong evidence of positive impact in the field of maternal and child health: evaluations have demonstrated increased frequency of growth monitoring, higher prevalence of overall and exclusive or predominant breastfeeding, and delayed introduction of bottle-feeding. Findings also suggest positive impact from interventions targeting infectious and chronic non-communicable diseases (NCDs), including a reduction in health inequities [3].

The Maria de Socorro Silva e Souxa Family Clinic is one of the three MoH clinics operating in Rocinha, the largest favela in Brazil. **(See table 5).**

**Table 5.** Key features of the Community Health Worker Program at Maria de Socorro Silva e Souxa Family Clinic

| **Maria do Socorro Silva e Souza Family Clinic (Brazil)** | |  |
| --- | --- | --- |
| **Coverage population** | 32,000 |  |
|  |  |  |
| **Number of CHWs** | 66 |  |
| **Selection criteria** | - Literate  - >18  years  - Resident of the community  - Satisfactory performance on a knowledge test  - Two interviews: first by the human resources staff and second, by the health center staff  - “Have a spirit of leadership and solidarity” |  |
| **Compensation per hour of work/training ($US)** | 2.07  US dollar (they work 140-160 hours per month) |  |
| **Key tasks** | - Provide directly observed therapy for TB and HIV patients  - Conduct regular home visits (between 1-2 every two weeks) for patients living with chronic conditions (diabetes, hypertension, mental illness, malnutrition)  - Conduct home visits to all families in their catchment area at least once every 3 months  - provide informal health promotion advice during home visits  - Organize physician and nurse agendas and serve as the receptionist at the clinic  - Update community’s demographic information  - remind patients about  their appointments at the clinic  - Deliver medications at home in special cases (e.g., a diabetic patient who runs out of insulin a week before he/she is to be seen in the clinic)  - Encourage sick patients, during home visits, to seek out healthcare services and make an appointment for them  - Guide and walk with the health staff to visit sick patients who cannot reach the clinic  - Participate in weekly meetings with the Family Health Team (FHT). In these meetings complex cases are discussed from an interdisciplinary perspective |  |
| **Best practices** | - The FHT has a defined geographical area to deliver all healthcare services (geographical accountability)  - 28% of the entire budget from the municipality is invested in Primary Health Care (PHC)  - PHC doctors working with the municipality, are better paid than other specialists (e,g., surgeons, internal medicine doctors)  - Doctors and nurses are encouraged and rewarded to collaborate and include CHWs in the care delivery  - Private civil organizations -- through a public-private contract -- are in charge of hiring new staff and infrastructure maintenance  - Performance indicators --including clinical control and patient satisfaction-- are reported to the FHT every three months. The FHT receive a bonus if they reach specific health targets  - New hired staff have to pass a 3 month probation period to be contracted |  |

**Source:** CHW program managers, implementers, CHWs and patients.

***Acompañantes* in Chiapas, Mexico**

*Compañeros en Salud* (CES) is the Mexican affiliate of Partners in Health (PIH). PIH is an international non-governmental organization with multi-national experience collaborating with CHWs across a broad range of medical conditions, including HIV-AIDS, mental illness, tuberculosis, NCDs (diabetes mellitus and hypertension), and maternal-child health. Since February 2012, CES has worked in partnership with the Mexican Ministry of Health to rehabilitate, staff, and operate existing government primary care clinics in rural communities in Chiapas, one of the poorest states in Mexico.

The *Acompañantes* program was founded to address the burden of NCDs in the area. *Acompañantes* are women from the communities who are trained to support patients suffering from chronic diseases such as diabetes, hypertension, and depression. The focus of the *Acompañantes* program is strengthening community capacity in resource-limited settings by including local members in the formal health care delivery value chain.

The *Acompañante* approach has improved a number of metrics: disease clinical control, adherence to medications, patients’ understanding of their diseases, and attendance to clinic appointments. Currently, the clinical control rates of NCDs at CES’ clinics improved after the introduction of the Acompañantes program and are better than those at the average clinic in Mexico [4] – an achievement that has been made possible by the *Acompañantes* as well as by other CES interventions such as continuous training and supervision of doctors, the provision of clinical tools (such as NCDs management algorithms and UpToDate@), and a reliable supply of medications in the clinics. **(See table 6).**

**Table 6.** Key features of a CES-supported Community Health Worker Program in Laguna del Cofre, Mexico

| **Health Center in Laguna de Cofre (Mexico)** | |
| --- | --- |
| **Coverage population** | 2,500 |
| **Number of CHWs** | 10 |
| **Selection criteria** | - Literate  - > 21 years  - Resident of the community  - Be nominated (by the community or by herself) during a community meeting  - Interview with the program coordinator  - Show a genuine willingness to serve the community |
| **Compensations per hour of work/trainings** | 2.03 US dollar (they work 25-30 hours per month); paid as a food package/food coupons redeemable at a government-run food program |
| **Key tasks** | - Dispense directly observed therapy for TB and HIV patients  - Conduct weekly home visits to patients with chronic conditions (e,g., diabetes, hypertension, and mental illness)  - Provide informal health promotion advice during home visits, and psychological support to patients with depression  - Remind chronic patients about their appointments at the clinic  - Escort patients with chronic diseases to their clinic appointments as needed (i.e., if the patient speaks a different language and needs translation support)  - Deliver medications at home in special cases (i.e., a diabetic patient runs out of medications a week before his/her appointment)  - Conduct home visits with the health staff, especially for patients who cannot reach the clinic  - Participate in monthly clinical meetings to discuss complex cases with the physician |
| **Best practices** | - Cross-community collaboration on initial trainings for new CHWs  - Yearly CHW social events keep CHWs from different communities connected  - There is a clear definition of accompaniment in the program design and implementation, and many of the structures and processes are aligned in that direction  - Clear delegation of responsibilities among the staff at the program  - Satisfaction surveys conducted monthly with patients, CHWs, supervisors, and physicians  - Supervisors are selected based on performance after 6 months on duty, at minimum |

**Source:** CHW program managers, implementers, CHWs and patients.

**Key Findings: assessing the programs using the Accompanimeter 1.0**

In the following paragraphs, we will give a general overview -- using the five building blocks -- to highlight common characteristics and specific differences among programs that allowed us to offer each program an assessment using the Accompanimeter 1.0. We conducted interviews and held focus groups with CHW program managers, implementers, CHWs, and community members.

**Supervising**

In the two programs, the process of capacity building exhibited two main goals: to strengthen the skills of the CHWs, and to establish accountability between CHWs and the health system as a whole.

*Strengthening CHWs’ skills*: the supervision process serves to maintain regular contact between the supervisor and the CHWs in order to reinforce practical skills during home visits. In CES, for example, the supervisor provides on-site advice to the CHWs when assisting with complex cases such as patients on multiple medications and suffering from low adherence.

*Establishing accountability:* This goal of supervision is reflected by the CHW program at CES and in Rio de Janeiro, where CHWs are required to check in each day at their assigned clinic before going out to perform their daily duties. In both programs, there are regular meetings (weekly for Brazil, monthly for CES) to deliver feedback about indicators, assign new patients, discuss particular cases, and clarify any doubts related to CHW duties or patients’ concerns.

In Brazil, the Family Health Team offers a structure of mutual accountability among health professionals (doctors, nurses, nutritionists) and the CHWs. In many cases, the outcome of the patient depends on each member of the Family Health Team playing his or her part, and this creates accountability between members of the FHT. An assigned nurse serves as the supervisor of the CHWs, and in this role she supports the CHWs in attending to complex cases. However, the CHWs referred to the weekly 3-hour meeting with the FHT as the actual supervision.

“*Every week, we have a meeting with all the Family Health Team, and we discuss all the complex cases with the nurses, doctors and social workers.”* (CHW in Maria do Socorro Silva e Souza Family Clinic, Brazil)

At CES, one CHW, after a trial period of 6 months of working as CHW, is chosen by his or her peers to be the community-level program supervisor. Community members and CHWs alike remarked on the importance of this person in giving the broader community a voice in cases where the program needed structural or thematic adjustments. The supervisor visits a minimum of one patient per CHW each month to assess patient satisfaction and to support the CHWs with complex cases.

*“The woman [supervisor] comes every two weeks to ask me how I feel, how am I doing with my Acompañante [CHW] and if the visits have been helpful.” (*Community member, Laguna del Cofre, Mexico)

In the two programs, the community appeared to have little to no input in identifying, suggesting, or making necessary improvements to the structure of the CHW program. In both contexts, the capacity building and supervisory role played by the community was minor. In both programs, patients felt they had a voice in only reporting underperforming CHWs, but not in other aspects of program input.

*“If the Acompañante (CHW) is doing a bad job, I can talk with the doctor and he can reprimand her.”* (Community member, Laguna del Cofre, Mexico)

*“I believe that if the CHW is not working I can tell the clinic personnel and they can reduce his/her salary.”* (Community member, Maria do Socorro Silva e Souza Family Clinic, Brazil).

**Partnering**

The two studied programs clearly recognize that the ability to interact with, and engage, other sectors to improve health is crucial, especially when addressing diseases that have a strong interplay with the social determinants of health, such as malnutrition, tuberculosis, and diabetes. However, based on our assessment, interactions between the health system and other non-health sectors were limited in the two programs.

The highest inter-sectoral collaboration was seen in Brazil, where CHWs have the ability to link patients to *Bolsa Familia*, a conditional cash-transfer program for marginalized populations. This link was reported as useful by some community members, especially in cases where illness (such as tuberculosis) was closely related to social exclusion.

*“I remember a case of a poor woman with tuberculosis. My role was to give her TB pills every day, but because she did not have any food […] I made sure she got into Bolsa Familia. Definitively, our job is more than health; it is a social commitment.”* (Community member, Maria do Socorro Silva e Souza Family Clinic, Brazil)

 Equally important were the Family Health Team meetings, wherein the CHWs could openly interact with a social worker that can further assist in connecting families to the national conditional cash transfer program, *Bolsa Familia*. However, no additional social programs were mentioned.

In the CES, Mexico, CHWs were valued by the community for their role to preserve health. However, the community and CHWs agreed the program had limited ability to link patients with resources from other sectors outside of health:

*“Here the community authorities do not make our work harder when we refer a patient to the hospital…[They] can help us organize some things, for example they may transport one of our patients to the clinic... besides that, we do not have more contact with other authorities or the schools, for example.”* (CHW, Laguna del Cofre, Mexico)

On the two programs, stronger partnerships could be established with non-health sectors to improve other domains that indirectly affect patients’ health outcomes. While health personnel and community members alike referred to such partnerships as desirable, executive program managers noted particular challenges inherent to intersectorality. Making health a social end-goal would require a re-definition of how other social services––like economic development programs and education––are to be delivered, and implies investment in networks of care across sectors instead of siloed, individual sector approaches.

**Incentives**

In the two programs studied, material and non-material incentives were provided to CHWs. In both programs, CHWs felt that their material incentives (salary, equipment, etc.) were too low. It seems their main incentive is related to an intrinsic desire to receive appreciation from the community they serve and effect social change for the younger generations:

*“[W]hen you receive the love of your patients, a smile, a thank you, or kind words, is when you say: wow, my work is worthwhile regardless of my salary. We do this for love of our communities, for the future of our children, otherwise I would never do it. The salary is low.” (*CHW, Maria do Socorro Silva e Souza Family Clinic, Brazil).

The most enabled CHWs were found in Brazil, where CHWs are recognized as municipality employees and receive an established salary 1.5 times the minimum wage (minimum wage is 10 dollars a day), performance incentives, vacation time, bonuses for high performance, and social insurance. Additionally, the CHWs in Brazil were expected to invest the most time in their work (approximately 35 hours per week).

At CES in Mexico, CHWs receive a monthly food package based on 3 times the minimum daily wage, which is 4 dollars per day. The total value of the food package is based on the total hours of work that a CHW spends in an average month. A benefit of CHW payment in CES is that in rural communities in this region there is a lack of remunerated work opportunities for women. A limitation is that these women receive food instead of money, so they aren't given an option to purchase other goods instead. Most CHWs referred to their work in the program as their first-ever formal job, which is likely an additional motivator.

*“I always wanted to work for my community, but I did not have the opportunity to do so. The program [Acompañantes] gave me that opportunity.”* (CHW, Laguna del Cofre, Mexico)

All the CHWs on both programs agreed that some regular material incentive would permit them to invest more time in their work, and allow them to contribute to their family’s income. Community members agreed that the CHWs must be remunerated for their work.

**Choosing**

The program coordinators felt that selecting the “right” CHWs was one of the most crucial elements for success. Across all programs, all participants from the executive to the community level agreed that even with the right incentives and working environment, there are certain “negative elements” that, if ignored in the selection process, can result in underperforming CHWs.

Of special note is the process used by Brazil to choose their CHWs, wherein private NGOs are in charge of hiring all the health personnel. They have a three-stage method: first, a written exam, followed by an interview with the recruitment staff. If the candidate passes these two stages, he or she will be interviewed by the clinic personnel and, if selected, will spend the first 3 months in a probationary period. Community members not currently serving as CHWs expressed an interest in becoming CHWs if the opportunity arose. This interest by community members in Brazil in becoming a CHW was also found within community members in the CES program in Mexico.

CES in Mexico communities nominate their CHWs in a general meeting. Community members also can nominate themselves. Potential candidates are then interviewed by the program coordinators who had created an adapted screening questionnaire that attempts to assess the applicant’s capacity for conflict management, community service, and respect for others’ opinions. The questionnaire had been tested in three communities with good results according to program managers, clinicians and supervisors assessed for this study. The attrition rate at CES has been less than 3% in the past 5-year period.

**Education**

On the two programs, CHWs and the community have little input in the topics incorporated in the CHW training curricula. The chosen topics are decided upon at a central level according to local epidemiological priorities, and then adapted and implemented from the regional offices.

In terms of pedagogy, role playing was deemed by program coordinators in both programs to be a successful, and culturally appropriate, teaching strategy because it was based on experiential learning. CHWs agreed this was a preferable method of learning compared to a lecture-based style because it was more fun:

*“We have a really good time during the training sessions because [they] are interesting and funny [….] Also, we have the opportunity to act in the role-plays.”* (CHW, Laguna del Cofre, Mexico)

The length of the initial training is variable in both programs in our study. In CES in Mexico, the CHWs are trained for an initial period of one month, followed by a regular continuous education program provided each month in which new concepts and skills are introduced, and previous ones reinforced. In Brazil, instead of a formal initial training the CHWs are introduced to their duties by their supervisors. This in-practice introduction was described by the CHWs as “not the best way,” given that it made difficult to understand the bigger picture of their role in the program. Furthermore, some CHWs in these programs felt unprepared and unsupported in conducting their first home visits:

*“In the past, the introductory training was much better. For example, I had an entire month of initial training. Now the CHWs are introduced to the work in 4 days or less and this is not enough time to learn all that we need to make good home visits.”* (CHW, Maria do Socorro Silva e Souza Family Clinic, Brazil)

For an visual assessment of the two programs using the Accompanimeter 1.0, see Figure 4 and Figure 5.

**References**

[1] Cornwall A, Shankland A. Engajamento com cidadãos: lições aprendidas na construção do Sistema Único de Saúde. Psicol. Em Rev. 2013;19:519–542**.**

[2] Mendonça MHM de, Matta GC, Gondim R, et al., editors. Atenção primária à saúde no Brasil: conceitos, práticas e pesquisa. Rio de Janeiro, RJ: Editora Fiocruz; 2018.

[3] Giugliani C, Harzheim E, Duncan MS, et al. Effectiveness of Community Health Workers in Brazil: A Systematic Review. J. Ambulatory Care Manage. 2011;34:326–338.

[4] Newman PM, Franke MF, Arrieta J, et al. Community health workers improve disease control and medication adherence among patients with diabetes and/or hypertension in Chiapas, Mexico: an observational stepped-wedge study. BMJ Glob. Health. 2018;3:e000566.
